# Development and characterization of mammary intraductal (MIND) spontaneous metastasis models for triple-negative breast cancer in syngeneic mice

**DOI:** 10.1038/s41598-020-61679-8

**Published:** 2020-03-13

**Authors:** Xu-Liang Luo, Lan Lin, Hui Hu, Fang-Ling Hu, Yan Lin, Man-Ling Luo, Lin Wang, Yuan-Qiao He

**Affiliations:** 1 Department of Breast Surgery, The Second Affiliated Hospital of Nanchang University, Nanchang, Jiangxi P.R. China; 2 Department of Laboratory Animal Science, Nanchang University, Nanchang, Jiangxi P.R. China; 3 Peking University Shenzhen Hospital, Shenzhen, Guangdong P.R. China; 4 Medical College of Nanchang University, Nanchang, Jiangxi P.R. China

**Keywords:** Cancer models, Experimental models of disease, Translational research, Breast cancer

## Abstract

Triple-negative breast cancer (TNBC) has a more aggressive phenotype and higher metastasis and recurrence rates than other breast cancer subtypes. TNBC currently lacks a transplantation model that is suitable for clinical simulations of the tumor microenvironment. Intraductal injection of tumor cells into the mammary duct could mimic the occurrence and development of breast cancer. Herein, we injected 4T1 cells into the mammary ducts of BALB/C mice to build a preclinical model of TNBC and optimized the related construction method to observe the occurrence and spontaneous metastasis of tumors. We compared the effects of different cell numbers on tumorigenesis rates, times to tumorigenesis, and metastases to determine the optimal number of cells for modelling. We demonstrated that 4T1-MIND model mice injected with 20,000 cells revealed a suitable tumor formation rate and time, thus indicating a potential treatment time window after distant metastasis. We also injected 20,000 cells directly into the breast fat pad or breast duct for parallel comparison. The results still showed that the 4T1-MIND model provides sufficient treatment time for lung metastases in mice and that it is a more reliable model for early tumor development. The 4T1-MIND model requires continuous improvement and optimization. A suitable and optimized model for translational research and studies on the microenvironment in TNBC should be developed.

## Introduction

Breast cancer is the most commonly diagnosed cancer type and the leading cause of cancer death among women worldwide^1,2^. Breast ductal carcinoma *in situ* (DCIS) accounts for 15%–30% of all breast cancer cases^3^. DCIS tends to break through the matrix and develop into invasive ductal carcinoma^4^. Approximately 90% of all breast cancer-related deaths are caused by metastasis^5,6^, the most frequent sites of which are the lung, bone, liver, and brain. Triple-negative breast cancer (TNBC) is remarkably heterogeneous in terms of the tumor microenvironment^7^. Preclinical models that can return the breast tumor microenvironment to its original condition play a pivotal role in studies on the pathogenesis and treatment of breast cancer. However, 23 anticancer drugs failed in clinical trials between 2007 and 2010^8^, partly because the preclinical models used to test them were inappropriate for breast cancer. These findings illustrate that the success of breast cancer research and treatment depends on the experimental animal model used.

Experimental animal models of breast cancer have been widely studied. Researchers should choose different animal models based on the needs of their experimental work. Today, the most frequently used breast cancer models include spontaneous breast cancer animal models^9–11^, induced breast cancer animal models^11–15^, allograft experimental animal mammary cancer models^16–19^, xenograft breast cancer models^20–24^, distant metastasis of breast cancer models^17,25–27^ and genetically engineered mouse models (GEMMs) of breast cancer^28–30^. Given the continuous developments in biotechnology and laboratory animal science, research methods on breast cancer have consistently improved. Hence, the selection of experimental animal models should be more stringent.

Animal models that can mimic the microenvironment of breast cancer are scarce, but many studies need such models. Some preclinical models are available for this purpose, but *in vivo* models reflecting the breast tumor microenvironment are limited. One advantage of genetically engineered mouse models of breast cancer is that the success rate of replicating the tumor is high, and the results are reliable. However, such models are time-consuming and costly to construct, and the site of the tumor is unpredictable^14^. The lack of a model that can mimic the tumor microenvironment and demonstrate spontaneous metastasis has hampered progress in understanding breast cancer evolution and therapy. Patient-derived xenograft models, another common tool, can capture disease heterogeneity; however, these models are impeded by their lack of an immune system. Hence, an excellent animal model of breast cancer is still needed; such a model should (1) clinically represent a certain type of breast cancer; (2) feature feasible and simple operations with wide applicability to laboratories; (3) mimic the breast cancer microenvironment and spontaneous metastasis; and (4) have an intact immune system.

The purpose of this article is to describe an animal model that can mimic the tumor microenvironment, demonstrate the spontaneous metastasis of breast cancer, and maintain a complete immune system. The developed model can reproduce the complete process of breast cancer occurrence and development. The cell lines used in this work should be annotated in the literature to obtain a better understanding of the characteristics of animal models of breast cancer. The 4T1 cell line represents TNBC, and over 1,000 studies have reported on the 4T1 cell line model. The mammary intraductal (MIND) model, in which cells are injected into the mouse milk duct system, can accurately simulate the microenvironment of breast tumors^31–34^. Studies on the 4T1-MIND model are limited^33^, but this animal model is highly suitable for breast cancer research. Hence, further studies are warranted. We searched for a suitable cell injection number to build a syngeneic MIND model and observe tumorigenesis and spontaneous metastasis. We have also generated animal models or optimized them on the basis of the original models, which is of great significance for the application of this model in breast cancer research.

## Results

### Intraductal growth and optimal cell number determination during development of the 4T1-MIND model

**Figure 1:**
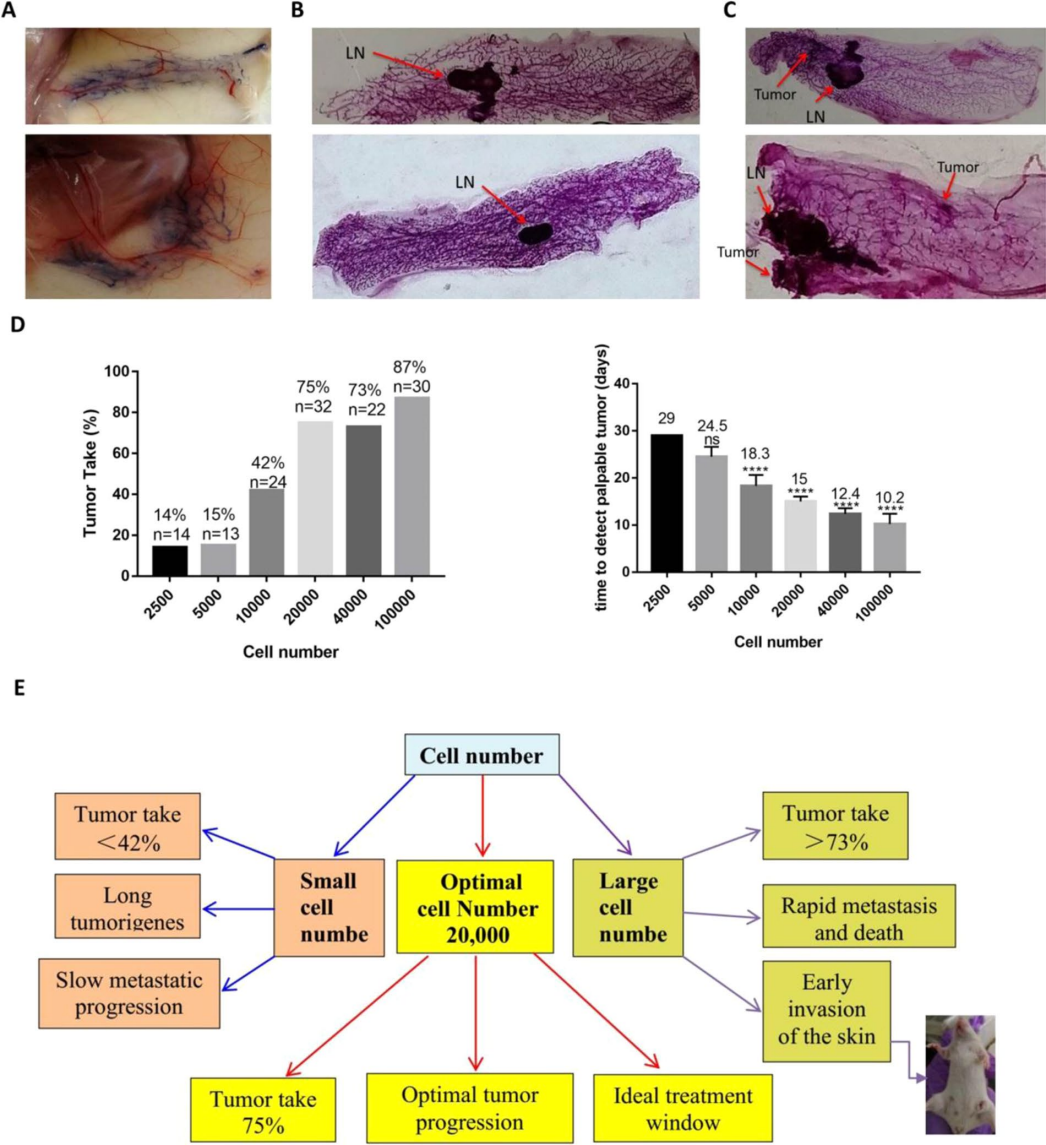
Determination of optimal cell number during development of the metastatic mouse model. (**A**) Representative photograph of injection of 0.2% trypan blue cells into the mammary glands of mice. (**B**) Representative photograph of the whole mount of the 4th inguinal mammary glands of female BALB/C mice injected with 20,000 4T1 cells for 1 week (*n* = 5). LN: lymph node. (**C**) Representative photograph of the whole mount of the 4th inguinal mammary glands of female BALB/C mice injected with 4T1 cells for 2 weeks (*n* = 5). LN: lymph node. (**D**) Rate of tumor formation and time of tumor formation after injection with different numbers of cells. ns. not significant, ****p < 0.0001. (**E**) Consequences of different numbers of injected cells.

The MIND model, in which cells are injected into the mouse milk duct system, can accurately simulate the microenvironment of breast tumors^31^. Cutting the nipple and injecting it with a cell suspension enabled observation of the suspension in the mammary duct (Fig. 1A). One to two weeks after injection, we observed the condition of the mammary duct, and the 4T1 cell line formed tumors in the mammary duct in the second week (Fig. 1B,C). We observed the engraftment rate of BALB/C mice that had been implanted with 2,500, 5,000, 10,000, 20,000, 40,000, or 100,000 4T1 cells. To characterize the latent dependency of tumor formation on cell number, we performed a cell titration of 2,500–100,000 4T1 cells. Tumors became palpable within 10 days in animals implanted with 100,000 cells, within 12 days in animals implanted with 40,000 cells, within 15 days in animals implanted with 20,000 cells, within 19 days in animals implanted with 10,000 cells, within 25 days in animals implanted with 5,000 cells, and within 29 days in animals implanted with 2,500 cells. The engraftment rate of different cell numbers in each group was between 14% and 87% (Fig. 1D). The tumor formation rate was high, and the tumor formation time was short in animals implanted with 100,000 and 40,000 cells; moreover, the transplanted tumor was likely to invade the skin (Fig. 1E). Model mice implanted with 10,000, 5,000, and 2,500 cells had low rates of tumor formation, which may be caused by the immune system. The results revealed that the optimal number of injected cells was 20,000. Next, we compared distant organ metastasis in each group.

### Visceral metastasis in the 4T1 MIND model

**Figure 2:**
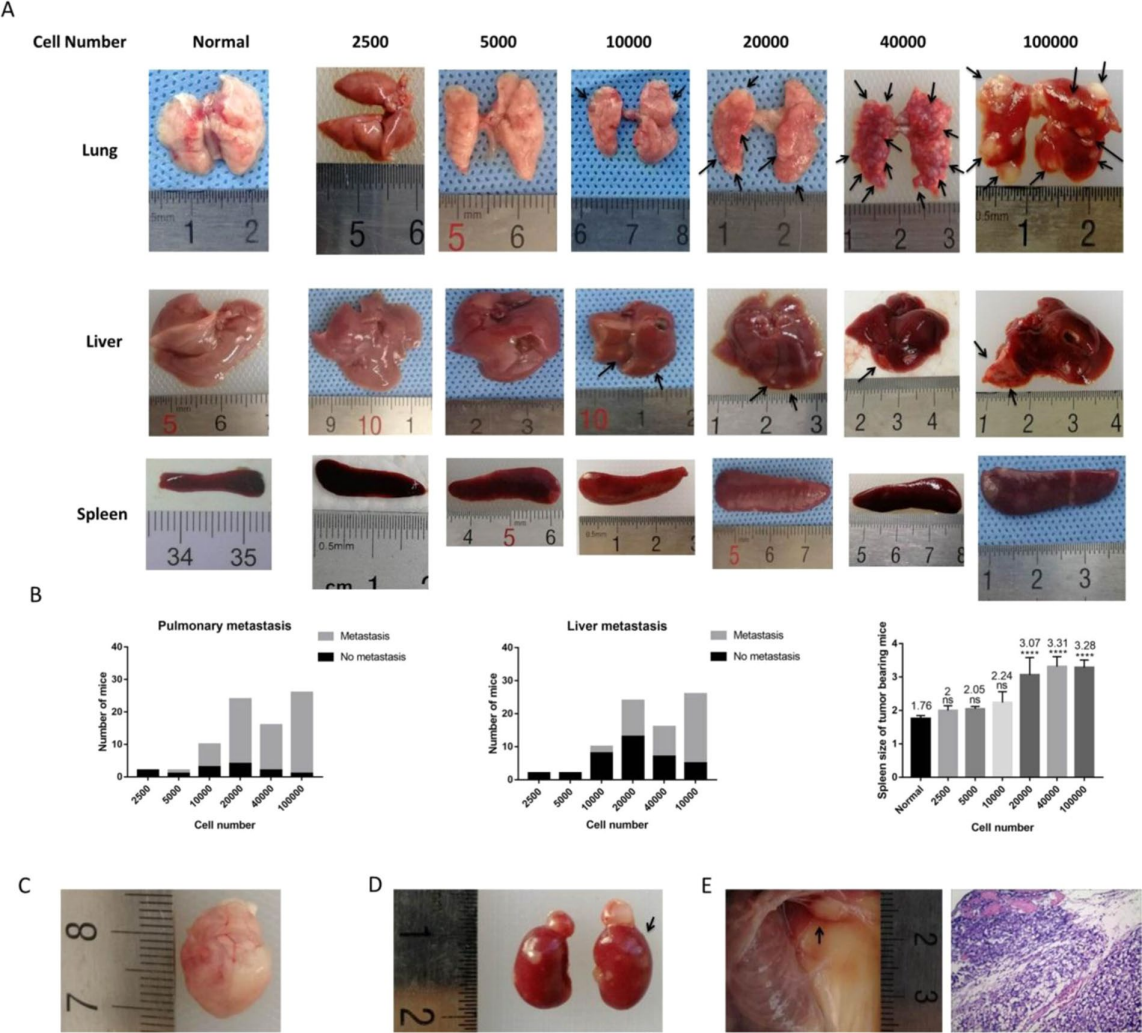
Visceral metastasis in the MIND model. (**A**) Representative photograph of the viscera of female BALB/C mice injected with each number of 4T1 cells for 7 weeks (n = 5). The size of lung metastasis and liver metastasis increased with the number of cells. Black Arrow: tumor. (**B**) The number of mice with lung or liver metastasis and the length of spleen in each group were compared. Lung metastases were found in nearly all mice implanted with 10,000, 20,000, 40,000, and 100,000 4T1 cells into their mammary duct. Mice injected with 5,000 cells only developed lung metastasis. The number of mice with liver metastasis increased with the number of cells. Compared with normal mice without injected cells, mice implanted with 20,000, 40,000, and 100,000 4T1 cells showed splenomegaly. (**C**,**D**) Brain and renal metastases are found in 4T1 MIND mice implanted with 100,000 and 40,000 4T1 cells. (**E**) The second pair of ipsilateral breast metastases was found in 4T1 MIND model mice that had been implanted with 100,000 4T1 cells. ****p < 0.0001, ns. not significant.

Approximately 7–8 weeks after injection, metastasis of distant viscera was found in the 4T1 MIND model (Table 1). We found multiple distant organ metastases in 4T1 MIND model mice that had been implanted with 100,000, 40,000, 20,000, and 10,000 4T1 cells into their mammary duct. Indeed, at weeks 7–8, macrometastases were mainly observed in animals injected with 40,000 and 100,000 cells, while micrometastases were observed in some animals injected with 10,000 and 20,000 cells. Large metastases were observed in the lungs and livers of mice injected with 100,000 and 40,000 cells, while most of the metastases in the lungs and livers of mice injected with 20000 and 10000 cells were relatively small (Fig. 2A). Lung metastases were found in nearly all mice implanted with 10,000, 20,000, 40,000, and 100,000 4T1 cells into their mammary duct. Mice injected with 5,000 cells only developed lung metastasis. The number of mice with liver metastasis increased with the number of cells. No lung or liver metastasis was observed in 4T1 MIND model mice that was implanted with 2,500 4T1 cells. Compared with normal mice without injected cells, mice implanted with 20,000, 40,000, and 100,000 4T1 cells showed splenomegaly (Fig. 2B). The most frequent sites of metastasis were the lung and liver, followed by the brain and kidney. Interestingly, we found rare cases of brain and renal metastases in 4T1 MIND model mice that had been implanted with 100,000, 40,000 and 20,000 4T1 cells (Fig. 2C,D). The second pair of ipsilateral breast metastases was found in 4T1 MIND model mice that had been implanted with 100,000 4T1 cells (Fig. 2E). Mice implanted with 40,000 and 100,000 4T1 cells developed multiple large metastases after approximately 7 weeks and were in poor condition. By comparison, mice implanted with 20,000 4T1 cells showed smaller metastases within approximately 7 weeks, but their condition was acceptable for at least another 2 or more weeks. This finding provides a potential time window for treatment after distant metastasis. Over 75% of the animals developed primary tumors by day 19 after injection with 20,000 cells, and micrometastases with a few cases of macrometastases could be observed at weeks 7–8. Injection of 20,000 cells produced an optimal model that could adequately represent all of the steps of metastasis while considering cost and time constraints. Thus, unless otherwise specified, all model constructions described hereafter are based on this optimized scheme. In subsequent sections, we used pathology to verify the feasibility of the model.

**Table 1:** Summary of sites of metastasis for 4T1-MIND^a^.

Cell number	Tumor Take^b^	Pulmonary metastasis^c^	Liver metastasis^c^	Brain metastasis^c^	Adrenal metastases^c^
2,500	2 (14)	—	—	—	—
5,000	2 (13)	1 (2)	—	—	—
10,000	10 (24)	7 (10)	2 (10)	—	—
20,000	24 (32)	20 (24)	11 (24)	3 (24)	1 (24)
40,000	16 (22)	14 (16)	9 (16)	2 (16)	1 (16)
100,000	26 (30)	25 (26)	21 (26)	4 (26)	4 (26)

^a^2,500, 5,000, 10,000, 20,000, 40,000 or 100,000 Cells were introduced into mammary duct of normal female BALB/C mice and dissected after 7 weeks as described in METHODS.

^b^Number of mice positive (total number of mice).

^c^Number of mice positive (total number of tumor bearing mice).

### Pathological features of tumors and visceral metastases in the 4T1-MIND model

**Figure 3:**
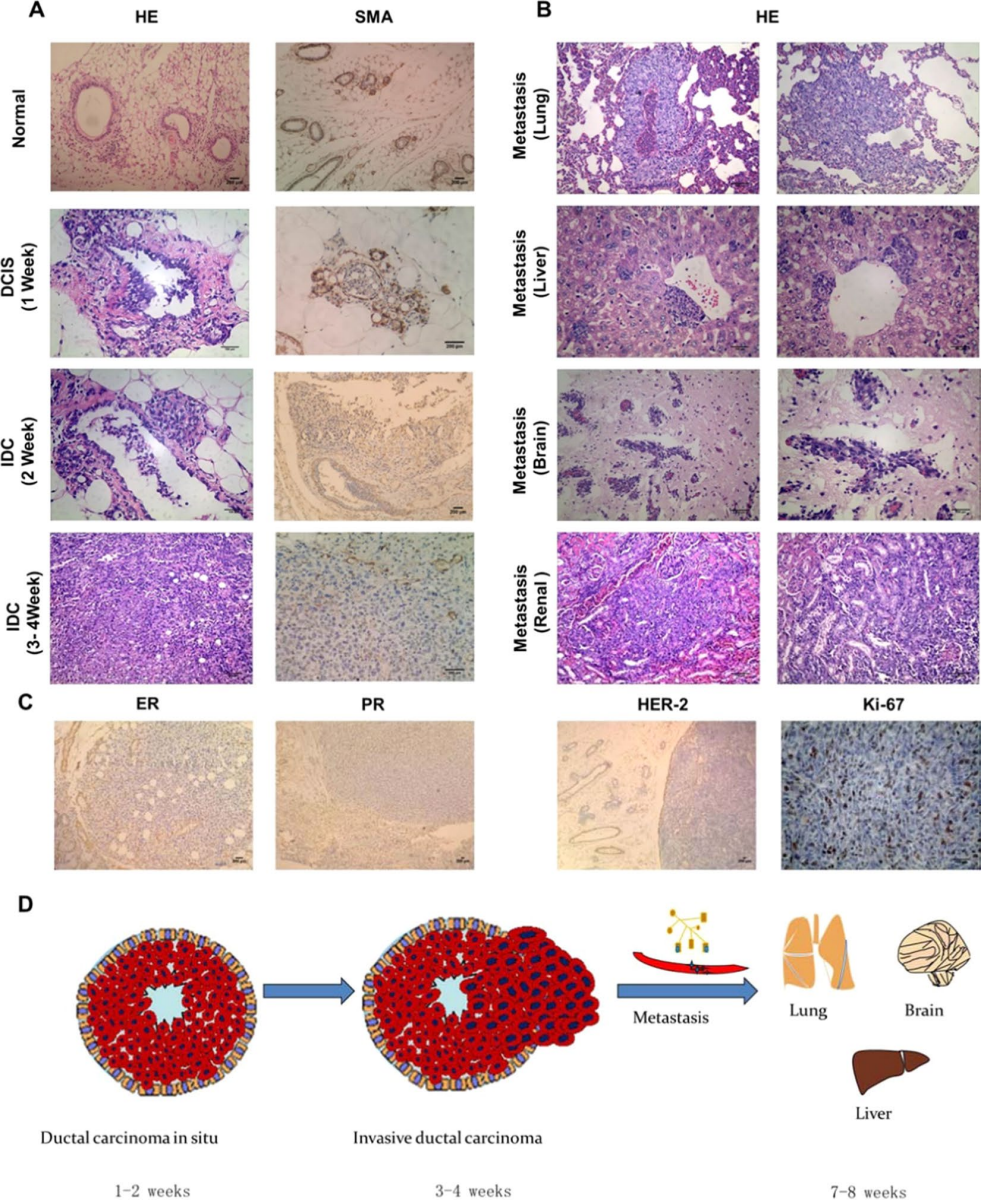
Pathological features of tumor and visceral metastases in the 4T1-MIND model. (**A**) Representative photographs of H&E staining, SMA immunohistochemical staining sections of normal ducts and the mammary glands of female BALB/C mice injected with 4T1 cells. The ductal architecture demarcated by SMA in the normal breast ducts (n = 5). In the first week, a DCIS-like structure formed in the female BALB/C mice injected with 4T1 cells (n = 5), SMA immunostained the myoepithelial layer around the DCIS. In the second week, Tumor cells have just broken through the breast duct and grow outside of them, SMA immunostained the Part of MECs around the breast ducts (n = 5). IDC-like structure formed in the female BALB/C mice injected with 4T1 cells for 3–4 weeks (n = 5), the MECs almost disappears and expression of SMA was negative. Scale bar, 100–200 μm. (**B**) Representative photographs of H&E staining sections of the viscera of female BALB/C mice injected with 4T1 cells for 7 weeks (n = 5). Lung, brain and renal metastasis could be observed. Tumor cell metastasis from the central vein forms liver metastasis. Scale bar, 100–200μm. (**C**) Representative immunohistochemical staining sections of the tumors of female BALB/C mice injected with 4T1 cells for 4 weeks (n = 5). The ER, PR and HER-2 immunostaining sections of transplanted tumor were negative (positive expression around normal mammary duct), but the expression of Ki-67 was positive. Scale bar, 100–200 μm. (**D**) Schematic diagram of model development.

The ductal network in the human breast and mouse mammary gland is composed of two essential layers of cells: luminal epithelial (LE) and myoepithelial cells (MECs)^35^. MECs play a vital role in the development of mammary glands and help maintain the tissue integrity of the breast^36^. MECs are also present in the normal, premalignant breast as well as in DCIS, but to invasive ductal carcinoma (IDC), the growth of MECs is hindered and gradually disappears^37^. Given the importance of MECs in breast cancer progression, we used H&E and SMA (a MEC marker) immunohistochemical staining to describe the progress of 4T1-MIND tumors. The H&E and SMA immunohistochemically stained sections of 4T1-MIND tissues after injection showed that the tumor cells grew in the mammary duct one week after injection and were in the stage of carcinoma *in situ*. Two weeks after the injection, the pathology showed that the tumor cells partially broke through the duct, which indicated that the tumor cells began to break through the duct during the first week to the second week. At 3–4 weeks after injection, the tumors developed into invasive breast cancer (Fig. 3A). The pathological structures of liver and lung metastases were similar to those of primary tumors. Liver metastases around the central vein and brain metastases growing along blood vessels could be observed (Fig. 3B). This finding indicates that cancer cells may be transmitted through blood, consistent with the clinical pathway of breast cancer metastasis. Brain and renal metastases were also found (Fig. 3B). Thus, these findings indicate that the model has spontaneous distant metastasis. The ER, PR, Her-2, and Ki-67 indices of the 4T1-MIND mice were close to those of TNBC^38^ (Fig. 3C). The development process of the model is shown in the schematic diagram (Fig. 3D).

### Tumor progression in the 4T1 MIND model

**Figure 4:**
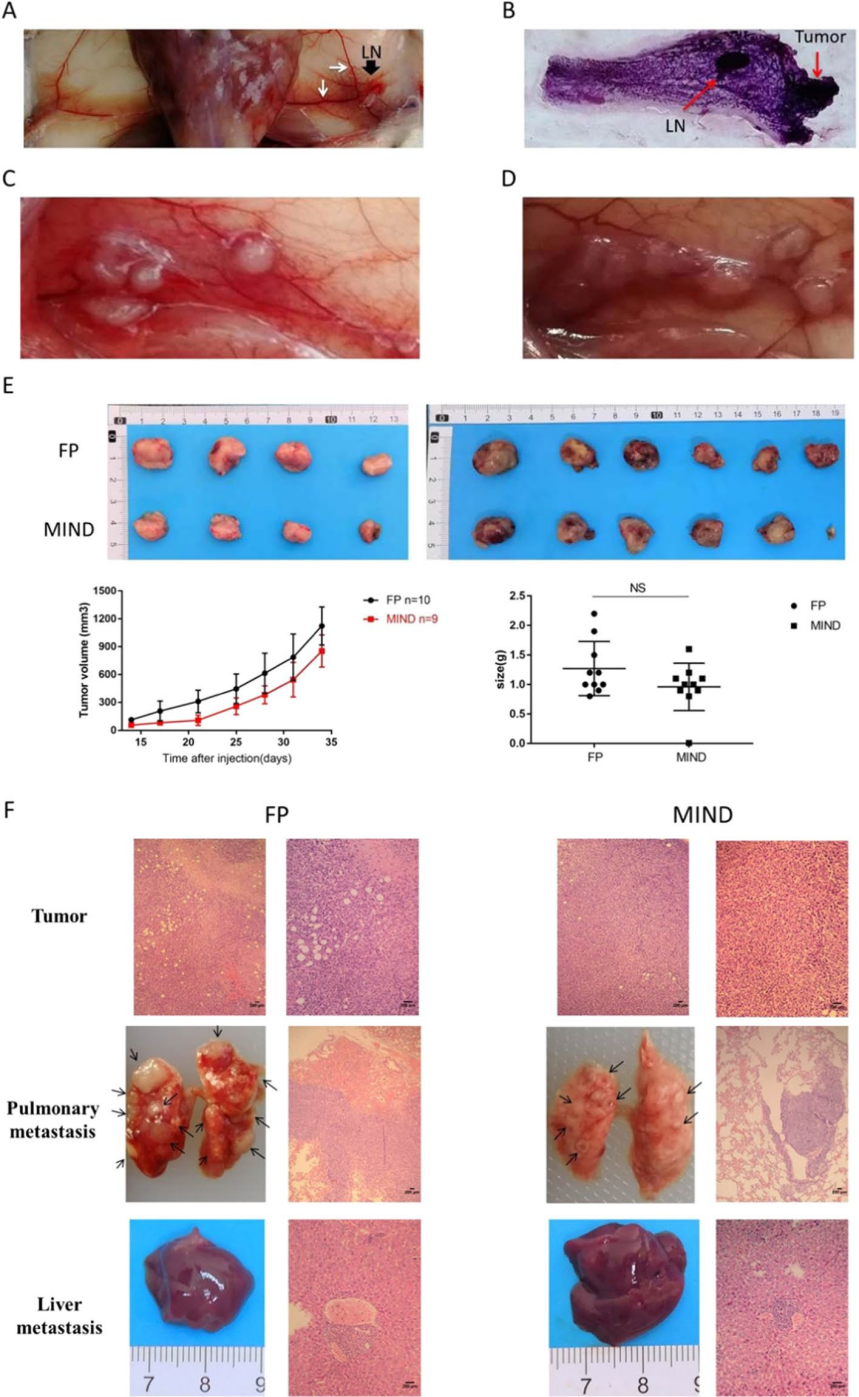
Tumor Progression in the 4T1 MIND Model. (**A**) Representative photograph of the 4th inguinal mammary glands of female BALB/C mice injected with 4T1 cells for 1 week (*n* = 5). Mammary ductal enlargement and lymphadenopathy and enlarged mammary vessels appeared. LN (black arrow): lymph node; white arrow: mammary vessels. (**B**) Representative photograph of the whole mount of the 4th inguinal mammary glands of female BALB/C mice injected with 4T1 cells for 2 weeks (*n* = 5). LN: lymph node. (**C**,**D**) Representative photograph of 4th inguinal mammary glands with tumor (T) of female BALB/C mice injected with 4T1 cells for three week (*n* = 5) and four week (*n* = 5). The photograph illustrates tumor growth in the mammary ducts. (**E**) Comparison of the two methods of tumor cell injection on primary tumor growth, following injection of 20,000 cells directly into the fat pad (FP) or via the MIND method. The growth curve and tumor weight of the two groups were compared 5 weeks after injection. (**F**) Representative photograph of transplanted tumor, lung metastasis and liver metastasis in female BALB/C mice of FP and MIND groups after injection of 20000 4T1 cells for 5 weeks. Scale bar, 200 μm.

We observed the occurrence and development of tumors in mice implanted with 20,000 cells. Exactly 1 week after intraductal injection of the mammary glands, duct dilatation was observed, and enlargement around the lymph nodes appeared. In the early stages of tumorigenesis, the vascular system flowing to the axillary lymph nodes begins to become engorged (Fig. 4A). Two weeks after the injection, tumor cells began to grow in the mammary duct to form tumors (Fig. 4B). Three to four weeks after the injection, the tumors continued to break through the mammary duct for further development. Then, the tumour status progressed to unlimited invasive ductal carcinoma (IDC). The tumor gradually covered the whole breast (Fig. 4C,D). In this model, the early shape of tumor formation was irregular and difficult to observe. We used MRI to confirm the formation and metastasis of the tumor (Supplementary Fig. S1). Next, we injected 20000 cells directly into the breast fat pad (FP) or via the MIND method for parallel comparison. The difference of tumor formation rate, metastasis range or metastasis site were compared (Table 2). The tumor formation rate of 20000 cells injected directly into the breast fat pad of mice was 100%, while that of mice injected into the mammary duct was 90%. However, the mice in the FP group were in poor physical condition due to heavy tumor load 5 weeks after injection, so they were euthanized 5 weeks after injection. The growth curve and tumor weight of the two groups at 5 weeks after injection are shown in Fig. 4E. The tumor mass in the MIND group was smaller than that in the FP group, but there was no statistical significance. There was no significant difference in pathological results between the two groups (Fig. 4F). However, the MIND model is a more reliable model for early tumor development. In the FP group, the tumor progressed too fast, and there were many metastases in the lung and liver. The mice in the FP group died too fast, and there was no suitable treatment time window. In the MIND group, more than half of the mice had small lung metastasis in the fifth week, which lasted at least seven weeks. This could provide sufficient treatment time for lung metastases in mice.

**Table 2:** Summary of sites of metastasis for 4T1-FP or 4T1-MIND^a^.

	Tumor Take	Pulmonary metastasis	Liver metastasis
FP	10 (10)^b^	10 (10)	5 (10)
MIND	9 (10)	7 (10)	3 (10)

^a^20,000 Cells were introduced into fat pads or mammary duct of normal female BALB/C mice and dissected after 5 weeks as described in METHODS.

^b^Number of mice positive (total number of mice).

^c^Number of mice positive (total number of tumor bearing mice).

## Discussion

In this work, we established a mouse model that is physiologically homologous to TNBC. This model provides a natural process for the growth of breast cancer, and the recovery of the microenvironment of breast cancer needs further study. In comparison with other existing preclinical models, the present model offers several advantages. First, the MIND model can better simulate the microenvironment of breast tumors and conform to the natural development of breast cancer. Second, it can restore spontaneous metastasis in breast cancer and has a complete immune system. The immune system plays an important role in the occurrence and development of tumors^39^. This model can be used to study the effect of immune responses on the development of breast cancer. Interestingly, this model easily led to spontaneous distant-organ metastasis, possibly due to the natural environment of breast cancer. The specific causes and processes of distant metastasis require further verification. The mouse model first develops ductal carcinoma *in situ*, which then breaks through the basement membrane and finally disseminates to distant organs. The development of this breast cancer model is similar to that of the MMTV-PyMT transgenic model, which reflects the multistage progression of human breast cancer from hyperplastic to spontaneous metastasis to metastasis in the lung^30^. The mammary myoepithelial cell layer acts as a defence against these tumor cells^40,41^. When a gap is present in the wall, intraluminal cancer cells break through the line of defence and transform into unconstrained IDC. Researchers at Johns Hopkins University recently proved that the myoepithelial layer around the mammary ducts can catch tumor cells and prevent them from spreading in the body^42^. The present model provides an opportunity for studying the development and metastasis of breast cancer. Previous works on distant metastasis depend on injecting a large number of tumor cells into circulation or specific organ sites^43,44^. Furthermore, the feeding conditions of BALB/C mice are not complex, and the operation method is easy; thus, operations can be carried out in most laboratories. The mechanics of organ metastasis should be further studied.

We showed that over 75% of the model animals develop primary tumors by day 19 after injection with the optimal number of 20,000 cells; micrometastases with some cases of macrometastases are also observed at weeks 7–8. Compared with the same number of cells injected into the breast fat pad model, the 4T1-MIND model has similar tumorigenesis and metastasis. In the FP group, the mice died because of rapid metastasis, and there was no suitable treatment time window. In the MIND group, more than half of the mice had small lung metastasis in the fifth week, which lasted at least seven weeks. This can provide sufficient treatment time for lung metastases in mice. The difference between the two groups may be due to the relative dispersion of tumor cells and the degree of immunity in the mammary duct. We chose the fourth pair of mammary ducts for injection because of their convenient location. Upon intraductal injection of mice, the direction of the injection was exactly the same as that of the mammary duct after lifting up of the skin of the nipple. In the initial cell titration experiment, primary tumor uptake was very low in animals implanted with 10,000, 5,000, and 2,500 cells because of their immune system. This finding may be applied to studies on the role of the immune system in cancer cells.Lymphocytes^45^, natural killer cells and macrophages can affect tumor growth^39,46–48^. We can use the proposed model to study the role of the immune system in tumor development. Finally, while we did not use the proposed model in drug tests, we believe it to be theoretically feasible and reliable for such tests. Drug validation of the model requires further experiments, such as irregular tumor measurement.

Taking these results together, we optimized an animal model with a complete immune system that can reproduce the spontaneous metastasis of TNBC. This model can be used to study the microenvironment of breast cancer, the development of spontaneous distant metastasis, and the concept of tumor-related immunity; it can also be used to intervene in the process of tumor development.

## Methods

### Cell culture

4T1 cells (ATCC® CRL2539™) were cultured in RPMI1640 medium and 10% foetal bovine serum in humidified 37 °C/5% CO_2_ incubators at either 2% or ambient O_2_ for 1–2 d. 4T1 cells of the logarithmic phase were trypsinized, centrifuged and suspended in RPMI 1640 medium. To prepare cells for injection implantation, the cells were pelleted at concentrations of 5,000,000 cells/mL, 2,000,000 cells/mL, 1,000,000 cells/mL, 500,000 cells/mL, 250,000 cells/mL and 125,000 cells/mL. Trypan blue (0.2%) was added to the cell suspension. The suspended cells remained at room temperature and were injected into the mouse within 30 minutes.

### Animals

Female BALB/C mice were purchased from the Nanchang Royo Biotech Co., Ltd. All the animals used in this study were 6–8-week-old virgin female BALB/C mice. Mice were housed in the animal room of Nanchang Royo Biotech Co., Ltd. Mice were fed commercial mouse diet food with a 12-hour light-dark cycle under pathogen-free conditions. At least five mice per experiment were used for this study. During the whole experiment, the animals were carefully monitored and euthanized if they showed symptoms including but not limited to reduced food or water intake, skin ulcers, hunched posture, weight loss, vocalization, irritability or lack of grooming. Euthanasia was performed using ether inhalation followed by cervical dislocation.

### Intraductal transplantation method

Recipients were 6- to 8-week-old virgin female BALB/C mice. Before transplantation, cells are suspended as single cells in RPMI1640 medium. A Hamilton syringe with a 50-μl capacity and a blunt-ended 1/2-inch needle was used to deliver the cells. BALB/C mice were anaesthetized using a mix of isoflurane and oxygen delivered by mask, the limbs were secured, the inguinal gland was sterilized and the inguinal nipple was exposed. The fourth nipple on the left side of the BALB/C mice was cut off so that the needle could be directly inserted into the mammary duct through the nipple. A Y-incision was made on the abdomen to expose the inguinal gland. The BALB/C mice were injected into the duct with 20 μl of cell culture medium containing suspended cells. These volumes filled the entire ductal tree^49^. The injected liquid could be visually detected in the duct. Then, the incision was sutured with silk thread, and the skin was disinfected.

### Grouping and construction of animal models

Twenty 6- to 8-week-old virgin female BALB/c mice were randomly divided into two groups, with 10 mice in each group. As described above, mice were anaesthetized, and a Y-incision was made on the abdomen to expose the fourth pair of mammary glands. In the FP group, 20000 4T1 cells were diluted with 100 microliters and injected into the fourth fat pad of mice with syringes. Then, the incision was sutured with silk thread, and the skin was disinfected. In the MIND group, 20000 4T1 cells were injected into the mammary ducts of the fourth pair of mice as described above. The tumor growth rate, metastasis range and metastasis site were observed. The long diameter (a) and short diameter (b) of the tumor were measured by callipers twice a week, the volume of the tumor was calculated according to v = ab^2^/2, and the growth curve was drawn.

### Mammary gland whole mount staining

The fourth mammary gland was dissected and spread on glass slides and fixed in Carnoy’s fixative (60% EtOH, 30% chloroform, 10% glacial acetic acid) at room temperature for 2–4 hours. The slides were washed with an alcohol/distilled water gradient, starting with 70% ethanol for 15 minutes, and then rinsed with distilled water for 5 minutes. Then, the mammary gland was stained in carmine alum (0.2% carmine, 0.5% aluminium potassium sulfate dodecahydrate) at room temperature (RT). The samples were washed in 70% EtOH for 15 minutes, washed in 95% EtOH for 15 minutes, and washed in 100% EtOH for 15 minutes. Samples were dehydrated and cleared in xylene for 2–3 hours and photographically recorded.

### Time course

BALB/C mouse ducts were implanted with 100,000, 40,000, 20,000, 10,000, 5,000 or 2,500 4T1 cells. The 4T1 cells were injected into the mouse for 1–2 weeks, and mammary gland whole mount staining revealed focally dilated milk ducts. Magnetic resonance imaging was used to confirm the transplanted tumor and detect whether there was distant organ metastasis in mice. The time of tumorigenesis was mainly reflective of the MRI results because the shape of the transplanted tumor was irregular and difficult to find through physical examination. After seven weeks, euthanasia was performed using ether inhalation followed by cervical dislocation (according to AALAS guidelines). BALB/C mice were dissected to observe lung, liver, kidney, spleen and brain metastases. The MRI was performed using a 1.0 Tesla whole body small animal MR scanner (Aspect M3, Israel). We used MRI to detect transplanted tumors and distant metastases. HE staining revealed morphological features of the transplanted tumor and distant organ metastasis. Immunohistochemistry was used to detect the expression of ER, PR, HER-2, Ki-67 and SMA in transplanted tumors. The pathology of tumor development in different stages in mice was analysed by director Li-Qing Wu, The First Hospital of Nanchang.

### Statistical analysis

Graph generation and statistical analysis were done on Prism 7.0 software (GraphPad, San Diego CA). All data are expressed as the mean ± SD. A value of P < 0.05 was considered statistically significant.

### Ethics approval

All animal studies were approved by the laboratory Animal Ethics Committee of Nanchang Royo Biotech Co., Ltd. Standard animal care and laboratory guidelines were followed according to the AALAS guidelines.

## Supplementary information

Supplementary Figure S1.

## Data Availability

The datasets generated and analysed during the current study are available from the corresponding author on reasonable request.
