# Supplementary material for: Development and characterization of mammary intraductal (MIND) spontaneous metastasis models for triple-negative breast cancer in syngeneic mice
Source: Sci Rep. 2020 Mar 13;10:4681. doi: 10.1038/s41598-020-61679-8 (PMC7070052; doi:10.1038/s41598-020-61679-8)
Supplement: Supplementary file 1 — Supplementary Figure S1. [file 41598_2020_61679_MOESM1_ESM.pdf]

Development and characterization of mammary intraductal (MIND) spontaneous metastasis models for triple-negative breast cancer in syngeneic mice

Xu-Liang Luo[1,4], Lan Lin[1,4], Hui Hu[3], Fang-Ling Hu[4], Yan Lin[4], Man-Ling Luo[4], Lin Wang*[1,4] and Yuan-Qiao He*[2]

[1]Department of Breast Surgery, The Second Affiliated Hospital Of Nanchang University, Nanchang University , Nanchang , Jiangxi , P.R. China.

[2]Department of Laboratory Animal Science , Nanchang University , Nanchang , Jiangxi , P.R. China.

[3]Peking University Shenzhen Hospital, Peking University , Shenzhen , Guangdong , P. R. China.

[4]Medical College of Nanchang University, Nanchang ,Jiangxi , P.R. China.

Lin Wang, 2287424928@qq.com

Yuan-Qiao He, heyuanqiao@ncu.edu.cn.

* Corresponding author.

Supplementary Figure S1.

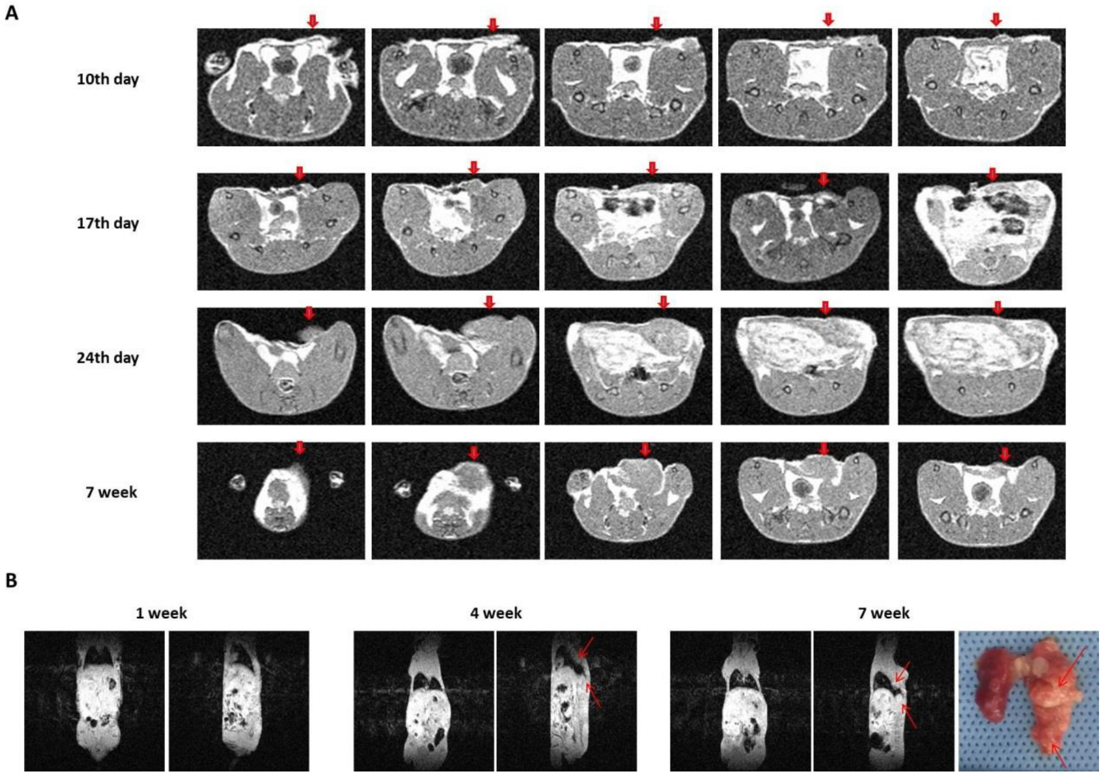

Supplementary Figure S1. Tumor Progression in the 4T1 MIND Model. (A) Representative MRI images obtained at different times after injection (*n* = 5). (B) Distant metastasis was detected by MRI in mice. At the 4th week, MRI indicated that there might be lung metastasis in mice. At week 7, MRI showed lung metastasis in mice, which was confirmed by anatomy.
